# Role of m^6^A mRNA Methylation in Plant Defense

**DOI:** 10.3390/epigenomes9040042

**Published:** 2025-10-15

**Authors:** Rakesh Srivastava, Niraj Lodhi

**Affiliations:** 1 Department of Biotechnology, School of Sciences, Noida International University, Noida 203201, India; 2 Department of Pathology and Genomic Medicine, Thomas Jefferson University, Philadelphia, PA 19107, USA

**Keywords:** epitranscriptomics, m^6^A methylation, plant immunity, biotic stress, fungus, bacteria, virus, insect, herbivore infestation

## Abstract

N6-methyladenosine (m^6^A) is the most abundant and dynamic RNA modification in eukaryotic messenger and non-coding RNAs, playing a pivotal role in the post-transcriptional regulation of gene expression. The coordinated actions of m^6^A writers, erasers, and readers influence transcript stability, immune activation, and pathogen suppression. Growing evidence indicates that m^6^A fine-tunes the expression of defense-related genes, modulates RNA processing events, and is frequently hijacked by pathogens and pests to promote virulence. Notably, the dual role of m^6^A in enhancing plant defense and facilitating pathogen adaptation highlights its significance in the host–pathogen arms race. This review emphasizes recent advances in our understanding of m^6^A-mediated epitranscriptomic regulation in plants, with a focus on its role in responses to biotic stresses, including fungi, bacteria, virus infections, insects, and nematode attacks. This regulatory layer offers novel opportunities for crop protection through targeted manipulation of the epitranscriptomic mechanism.

## 1. Introduction

Biotic stress caused by pathogens, insect pests, nematodes, and parasitic plants poses a significant threat to plant development and agricultural productivity. These stresses impair vital physiological processes, starting with poor seed germination and seedling establishment due to soil-borne pathogens and nematodes. Biotic stresses severely affect plant growth and development by damaging meristems, reducing chlorophyll levels, and impairing photosynthesis. They disrupt hormonal homeostasis, cause morphological deformities, and induce tissue necrosis and premature cell death. Reproductive organs are particularly vulnerable, often leading to flower and fruit abortion, poor grain quality, and reduced yields [1,2,3]. The cumulative effect of these disruptions can result in yield losses ranging from 20–40% globally, with severe outbreaks, such as rice blast, late blight of potato, or wheat rust, causing up to 80–100% crop failure [4,5]. The emergence of new pathogen strains, pesticide resistance, and climate change-driven shifts in pest populations further exacerbate the problem. Therefore, effective management of biotic stress is essential for sustaining plant health, ensuring yield stability, and maintaining global food security.

Plants counter biotic stress through complex defense mechanisms that involve innate immunity, including PAMP-triggered immunity (PTI) and effector-triggered immunity (ETI), the production of defensive metabolites, hormone signaling, and genetic and epigenetic regulation [6,7,8]. Upon pathogen or pest recognition, plants rapidly reprogram their transcriptome to activate defense-related genes such as pathogenesis-related (PR) proteins, enzymes for phytoalexin biosynthesis, and regulators of hormonal pathways [9,10,11]. The gene expression profile of a cell establishes its identity and functional status, emphasizing the necessity of cellular homeostasis. However, in reaction to environmental cues and developmental signals, cells must also be able to rapidly and precisely reprogram gene expression. This phenomenal adaptability is made possible by sophisticated, multilevel regulatory networks that provide eukaryotic cells with the flexibility and precision required to maintain functional integrity while adapting to changing physiological conditions [12,13]. Regulation occurs across multiple interconnected levels, including epigenetic, transcriptional, and post-transcriptional mechanisms that together ensure precise control over mRNA production, processing, and utilization [1,14,15,16,17]. Central to this regulation is cellular metabolism, a complex and responsive network of biochemical reactions that sustains homeostasis and adapts to fluctuating internal and external environments. The concept of metabolic reprogramming encompasses a broad range of physiological and pathological processes, reflecting the inherent plasticity of cells under stress [13,16,18]. Epigenetic modifications such as DNA methylation and histone modifications regulate gene expression by altering chromatin structure without changing the underlying DNA sequence [19,20,21]. Transcriptional regulation depends on the coordinated interaction between transcription factors and cis-regulatory elements, including promoters, enhancers, and silencers, which modulate RNA polymerase II recruitment and transcription initiation [14,15,17,22,23,24,25,26]. Beyond transcription, post-transcriptional mechanisms such as alternative splicing, RNA capping, polyadenylation, editing, nuclear export, localization, and degradation further refine mRNA fate and function [27,28]. Adding further complexity, chemical modifications to RNA molecules influence RNA stability, translation efficiency, and turnover [29,30].

Accumulating evidence suggested that RNA modifications have gained attention as an additional regulatory layer, influencing mRNA fate and expression. These reversible, non-genetic modifications shape the “RNA epigenome” or “epitranscriptome,” fine-tuning transcript functions [31,32]. RNA modifications are present across all domains of life, including Archaea, Bacteria, Eukarya, and viruses, with over 170 distinct modifications identified in various RNA types [33,34]. While their functions remain largely unknown, modifications in mRNAs are now well-documented in eukaryotes. This review intends to address RNA modifications, with a particular focus on N6-methyladenosine (m^6^A), in the context of pathogen and pest infestation. It highlights current insights, emerging directions, and key challenges in understanding the mechanistic roles of RNA modifications in plant responses to biotic stress, with implications for advancing crop resilience and sustainable agricultural practices.

## 2. Insights into m^6^A mRNA Modifications in Plants

Epitranscriptomics, much like DNA and histone modifications, modulates gene expression without making any changes to the actual mRNA sequence. The regulation of m^6^A marks dynamically shapes RNA fate, enabling precise control of stress responses and developmental processes. The m^6^A is a prevalent chemical modification involving the methylation of the nitrogen at the sixth position of adenosine within RNA molecules. The functional role of the m^6^A signature is predominantly mediated through its interaction with RNA-binding proteins, which recognize and interpret this modification. As a major post-transcriptional regulatory mark, m^6^A plays a central role in controlling gene expression. It is both dynamic and reversible, influencing various aspects of RNA metabolism, including transcript stability, alternative splicing, nuclear export, and microRNA-mediated regulation [35,36]. The evolutionary conservation of m^6^A across species, coupled with its responsiveness to environmental stimuli, positions it as a key regulator of gene activity and cellular adaptation.

**Figure 1:**
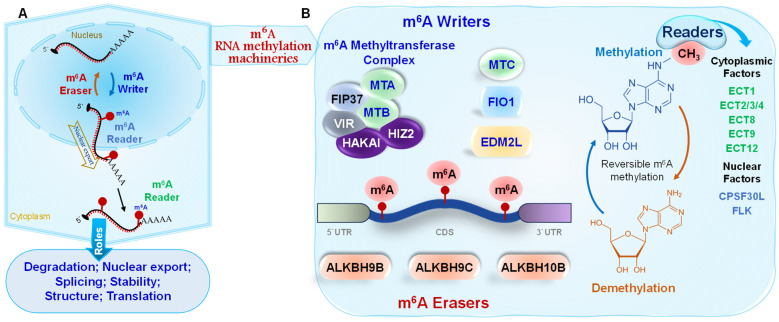
(**A**). The primary mechanism of m^6^A epitranscriptomic control, which dynamically control RNA fate throughout development and stress in plants. (**B**). The components of m^6^A mRNA methylation include writers (methyltransferases), erasers (demethylases) and readers (cytoplasmic and nuclear factors).

In plants, the core mechanism of m^6^A-based epitranscriptomic regulation operates as a dynamic and reversible process, governed by three protein groups: writers (methyltransferases) that deposit the mark, readers (RNA-binding proteins) that decode it, and erasers (demethylases) that remove it. In *Arabidopsis thaliana*, m^6^A methylation is catalyzed by a conserved writer complex [37] (Figure 1). Central to this complex are Methyltransferase A (MTA) and MTB, homologous to mammalian METTL3 and METTL14, respectively. These catalytic subunits are supported by auxiliary proteins, including Fkbp12-Interacting Protein 37 (FIP37; analogous to WTAP), Virilizer (VIR; similar to VIRMA), the E3 ubiquitin ligase HAKAI, and Hakai-Interacting Zinc Finger Protein 2 (HIZ2). These cofactors contribute to the stability and functionality of the writer complex [37,38]. Genetic analyses reveal that loss-of-function mutations in most writer complex components result in severe developmental defects such as impaired embryo formation and decreased seed viability. These phenotypes suggest the essential role of m^6^A in plant growth, development, and reproduction [39,40]. Beyond the canonical writer complex, an alternative m^6^A methyltransferase known as FIONA1 (FIO1), the Arabidopsis ortholog of METTL16, has been identified. FIO1 functions independently of the MTA–MTB complex and primarily targets small nuclear RNAs (snRNAs) and specific subsets of mRNAs [38,41]. In rice, OsEDM2L (Enhanced Downy Mildew 2-Like) has been identified as m^6^A methyltransferase. Mutation of *OsEDM2L* reduces global m^6^A levels, delays tapetal programmed cell death, and disrupts pollen formation by altering m^6^A modification, splicing, and polyadenylation [42]. In addition, *Solanum lycopersicum* SlMTC, a Class C MT-A70 family member related to AtMTC in Arabidopsis and METTL4 in humans, functions as m^6^A writer [43] (Figure 1). SlMTC interacts with SlMTA, and its knockout alters seed traits, reduces salt tolerance, and disrupts auxin signaling in tomato, indicating a key role in growth and stress responses [43]. Recruitment of the m^6^A methyltransferase in plants is guided by specialized proteins, particularly RNA-binding proteins and associated cofactors, which target the m^6^A writer to specific RNAs for context-dependent methylation. These recruiters ensure spatial and temporal precision of m^6^A deposition in response to developmental and environmental signals [37]. For example, Arabidopsis FCA (Flowering Control Locus A) directs m^6^A writer on the antisense transcript COOLAIR during flowering [44]; CRY2 (cryptochrome 2) under blue light executes liquid–liquid phase separation to recruit the m^6^A writer complex to circadian clock gene transcripts [45]; and rice FAP1 (FIP37-assocated protein 1) recruits OsFIP37 to facilitate methylation of YUCCA3 gene during male meiosis [46]. Beyond RBPs, chromatin state may also influence recruitment, as epigenetic marks such as H3K36me2 are associated with m^6^A deposition on nascent transcripts [47].

The transcriptome-wide m^6^A mapping has revealed that while m^6^A sites are distributed across the 5′ UTR and coding sequence (CDS), they are most strongly enriched near stop codons and within 3′ UTRs [40,48]. Despite species- and tissue-specific variation, enrichment of m^6^A near stop codons and within 3′ UTRs represents an evolutionarily conserved feature [48,49]. These sites largely conform to the consensus motif RRACH (R = A/G; H = A/C/U), but additional motifs such as URUAY (Y = C/U), UGUAW (W = U/A), and plant-specific motifs like URUAH have also been identified [40,50,51,52,53,54]. In Arabidopsis, FIONA1 targets hairpin-structured RNAs containing the UACAGAGA motif [55,56]. An MeRIP-seq study in wheat further reported enrichment of the GAACU sequence within m^6^A peaks [57]. Importantly, the functional outcomes of m^6^A depend on its location: modifications within the 5′ UTR often enhance translation, whereas those in the 3′ UTR primarily stabilize transcripts. In wheat, m^6^A deposition in the start codon and 5′ UTR enhances translation efficiency [58], while in apple (*Malus domestica*), MdMTA regulates 5′ UTR methylation of *Md4CL3* [59]. Also, over 60% of m^6^A sites occur at start codons of chloroplast-associated proteins and several photosynthesis-related genes, highlighting a role in photosynthetic regulation [50,60].

Once added, m^6^A marks exert their effects largely through “reader” proteins (Figure 1). In plants, the principal readers are YTH domain containing proteins that bind m^6^A-modified transcripts and modulate posttranscriptional fates such as stability, splicing, translation, and decay. *A. thaliana* encodes 13 YTH proteins, 11 belonging to the cytoplasmic YTHDF (ECT1 to ECT11) subfamily and two classified in the YTHDC group [61,62,63]. Comparative genomics reveals that the expansion of YTH domain proteins is conserved across plant species, with rice encoding 12 proteins and tomato 9 proteins [62,64]. Beyond the ECT readers, nuclear factors also recognize m^6^A modification such as the YTH domain containing isoform CPSF30L links m^6^A recognition to abscisic acid mediated stress signaling by modulating alternative polyadenylation [65]. Likewise, FLOWERING LOCUS K (FLK), the Arabidopsis ortholog of human IGF2BP proteins, binds m^6^A-modified FLC mRNA via its KH domains, regulating flowering time in an m^6^A dependent mode [66].

Alpha-ketoglutarate-dependent hydroxylase (ALKBH) family proteins, homologous to α-ketoglutarate-dependent dioxygenases, mediate m^6^A demethylation in plants [63] (Figure 1). In Arabidopsis, demethylases such as ALKBH9B, ALKBH9C, and ALKBH10B have been characterized using mutant analyses [63,67]. ALKBH family members have been identified as m^6^A demethylases in various crop species, playing key roles in stress responses and development. In tomato (*Solanum lycopersicum*), ALKBH2 was shown to regulate m^6^A levels and influence fruit ripening [68]. In rice (*Oryza sativa*), ALKBH9 contributes to male fertility regulation [69]. In cotton (*Gossypium hirsutum*), ALKBH10 has been implicated in salt tolerance [70]. While the mechanisms guiding the recognition and removal of m^6^A by these enzymes are not fully understood, they are thought to be crucial for the precise regulation of gene expression. Importantly, m^6^A is not limited to messenger RNAs; it also occurs in various non-coding RNAs, highlighting its broad regulatory roles and functional versatility [71,72]. Collectively, the coordinated actions of m^6^A writers, demethylases, and readers establish a flexible epitranscriptomic framework that fine-tunes gene expression during plant growth and stress adaptation.

In plants, DNA N6-methyldeoxyadenosine (DNA m^6^A) and RNA m^6^A represent two distinct adenine methylation events with important regulatory significance. DNA m^6^A is modification in which adenine is methylated at the nitrogen-6 position [73]. It is primarily enriched in gene bodies, promoters, transcription termination sites, and transposons, with reported sequence motifs such as ANYGA and GAGG, although motif preferences differ among species [74]. In Arabidopsis, AtMETTL4 has been identified as a methyltransferase catalyzing m^6^A deposition, while AtALKBH1A and AtALKBH1D act as demethylases; however, functional reader proteins remain unidentified [75,76]. Functionally, DNA m^6^A is often linked with active transcription when located within gene bodies by influencing chromatin accessibility, nucleosome positioning, and DNA–protein interactions, though it may act repressively when present in promoters [74]. Unlike RNA methylation, DNA m^6^A is relatively stable and can be inherited through both mitotic and meiotic divisions. Its abundance is also dynamically modulated under stress conditions, where it contributes to the regulation of stress-responsive genes, although the correlation between its presence and transcriptional outcomes is not always straightforward. Overall, DNA m^6^A is emerging as a regulatory epigenetic mark associated with chromatin dynamics and transcriptional control, though its biological roles in plants are still being elucidated through genome-wide profiling studies [77]. By contrast, RNA m^6^A constitutes a highly dynamic and reversible epitranscriptomic modification that governs post-transcriptional processes such as mRNA stability, splicing, translation, and decay [78]. Despite their chemical similarity, DNA m^6^A and RNA m^6^A operate at distinct molecular layers and timescales: the former provides a relatively stable means of transcriptional and chromatin-level regulation, while the latter serves as a rapid and reversible fine-tuner of gene expression. Together, these complementary modifications equip plants with multilayered regulatory strategies to coordinate gene expression during development and in response to environmental stress.

## 3. m^6^A RNA Modifications During Fungal Infection

**Figure 2:**
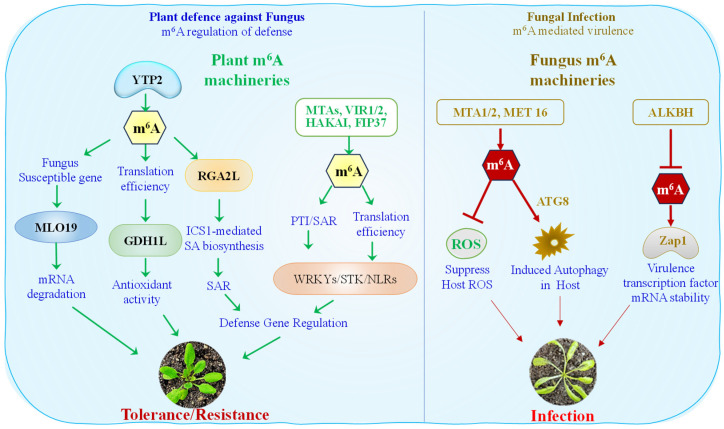
The figure highlights the epitranscriptomic tug-of-war between plants and fungi, where m^6^A modifications act as crucial regulators determining whether the outcome is plant resistance or successful fungal infection. This figure illustrates how m^6^A RNA methylation regulates plant–fungus interactions by influencing both plant defense and fungal virulence. On the plant side, m^6^A machinery components such as MTAs, VIR1/2, HAKAI, FIP37, and YTP2 enhance defense responses by regulating translation efficiency and stabilizing resistance-related transcripts. For instance, m^6^A modification leads to the degradation of the susceptible gene MLO19, promotes GDH1L expression to enhance antioxidant activity, and stabilizes RGA2L, which drives ICS1-mediated salicylic acid biosynthesis and systemic acquired resistance (SAR). Additionally, the regulation of key defense genes such as WRKYs, STKs, and NLRs strengthens both pattern-triggered immunity (PTI) and SAR, resulting in improved tolerance or resistance. In contrast, fungal m^6^A machinery, including MTA1/2, MET16, and ALKBH, facilitates infection by suppressing host ROS production, inducing host autophagy through fungal gene *ATG8*, and stabilizing the mRNA of virulence such as *Zap1* transcription factor. These processes enhance fungal pathogenicity and weaken plant defenses.

Plants defend against fungal pathogens through PTI and ETI. PTI involves recognition of fungal molecules like chitin, activating defenses such as ROS, MAPKs, and callose deposition. ETI uses resistance (R) proteins to detect effectors and trigger hypersensitive responses [3,79]. Hormones, antifungal compounds, and epigenetic regulation further enhance resistance. m^6^A is increasingly recognized as a pivotal regulator of plant immune responses against fungal pathogens (Figure 2).

Recent studies have demonstrated that plants deficient in m^6^A modification exhibit enhanced resistance to fungal infections, emphasizing its immunomodulatory role. For example, Arabidopsis *mta* mutants showed stronger resistance to *Botrytis cinerea*, suggesting that m^6^A modulates defense signaling and restricts pathogen invasion [80]. In *Arabidopsis*, single-nucleotide resolution m^6^A profiling during *Hyaloperonospora arabidopsidis* (*Hpa*) infection revealed a pathogen-induced global reduction in m^6^A levels, particularly affecting immunity-related transcripts. The E3 ubiquitin ligase *hakai-1* (subunit of methyltransferases complex) mutant, which exhibited ~70% overlap with m^6^A-loss patterns seen during infection, showed constitutively elevated basal defense gene expression and enhanced resistance to *Hpa* [81]. In apple (*Malus domestica*), nanopore direct RNA sequencing uncovered a regulatory link between sorbitol metabolism and m^6^A-mediated resistance to *Alternaria alternata*. Sorbitol-responsive methyltransferases MdVIR1 and MdVIR2 stabilized key defense transcripts *MdWRKY79* and *MdNLR16* (Nucleotide binding/leucine-rich repeat) via m^6^A modification, enhancing both their expression and translational efficiency [82].

Moreover, m^6^A epitranscriptomic regulation is emerging in cereal crops. In wheat (*Triticum aestivum*), infection by *Puccinia striiformis* f. sp. *tritici* (*Pst*) led to transcriptome-wide m^6^A profiling that revealed significant hypermethylation of upregulated defense-related genes, coinciding with transcriptional upregulation. Conversely, photosynthesis-related genes were hypomethylated and downregulated, potentially facilitating *Pst* pathogen colonization. qRT-PCR analysis revealed dynamic expression of m^6^A regulators during *Pst* infection in wheat. *TaALKBH11B* was upregulated in early infection, while *TaALKBH4B* peaked during colonization. Reader genes (*TaECT25*, *TaECT31*, *TaECT21*) were downregulated at 1 dpi, possibly due to reduced activity of writer genes (*TaVIR-D*, *TaVIR-A*, *TaHAKAI1-A*). Notably, *TaFIP37-2D* showed continuous induction, suggesting a central role in m^6^A-mediated defense [83].

Fire blight infection by *Erwinia amylovora* in pear triggers extensive m^6^A epitranscriptomic remodeling, with over 97,000 m^6^A peaks identified and nearly 3000 being infection-specific. These modifications positively correlated with transcript abundance, particularly in key immune genes involved in PTI and Systemic Acquired Resistance (SAR) pathways, such as *WRKYs* and serine/threonine kinases. Notably, m^6^A writers were downregulated post-infection, suggesting dynamic regulation of the methylation machinery. Coexpression and protein–protein interaction analyses revealed tightly linked networks among m^6^A writers (*MTA*), erasers (*ALKBH5B*), and readers (*ECT9*), with *ALKBH5B* and *ECT9* positively associated with immune signaling. Hypomethylated genes were enriched in metabolic and developmental pathways, while m^6^A-upregulated genes were enriched in immune-related functions. These findings highlight a crucial role of m^6^A modifications in stabilizing defense-related transcripts and orchestrating the pear immune response to *E. amylovora* [84].

Overexpression of the *Malus hupehensis* gene *MhYTP2*, a homolog of the Arabidopsis m^6^A reader *ECT2*, in *Malus domestica* significantly enhanced resistance to *Podosphaera leucotricha*. *MhYTP2* modulated m^6^A regulatory genes by downregulating writers (*MdMTA*, *MdMTB*, *MdFIP37*) and the eraser *MdALKBH6*, suggesting altered mRNA methylation. This enhanced resistance was associated with downregulation of the susceptibility gene *MdMLO19* and its isoform *MdMLO19-X1*, likely via m^6^A-mediated mRNA degradation, and increased translation efficiency of the antioxidant gene *MdGDH1L*, leading to elevated protein levels and antioxidant activity. These results link m^6^A regulation to enhanced apple disease resistance through modulation of susceptibility and antioxidation pathways [85]. Interestingly, MhYTP2 influences both m^6^A-modified and non-methylated RNAs, as shown by its role in stabilizing the non-m^6^A-modified NBS-LRR resistance gene *MdRGA2L*, which confers protection against Glomerella leaf spot (GLS) caused by *Colletotrichum fructicola*. The m^6^A reader gene *MhYTP2* negatively regulates GLS resistance in *M domestica*. Upon infection, *MhYTP2* binds directly to the mRNA of *MdRGA2L,* reducing its stability and expression. *MdRGA2L* enhances resistance by promoting *ICS1*-mediated salicylic acid (SA) biosynthesis, triggering systemic acquired resistance. Overexpression of *MhYTP2* suppresses this pathway, compromising defense. Thus, *MhYTP2* acts as a negative regulator of GLS resistance, and *MdRGA2L* represents a valuable target for breeding disease-resistant apple varieties [86].

Beyond their regulatory functions in plant hosts, m^6^A RNA modifications have emerged as critical regulators of virulence and developmental processes in phytopathogenic fungi and oomycetes (Figure 2). In *Magnaporthe oryzae*, the rice blast fungus, MTA1 is essential for m^6^A RNA methylation and autophagy. Loss of *MTA1* disrupts m^6^A deposition, impairs autophagic activity, and significantly reduces fungal virulence. MeRIP-seq revealed 659 hypomethylated peaks affecting 595 transcripts, including *MoATG8*, whose mutation resulted in autophagy failure and attenuated pathogenicity [87]. In the oomycete *Phytophthora sojae* relies on the m^6^A methyltransferases *PsMTA1*, *PsMTA2*, and *PsMET16* for successful infection. Mutants lacking these genes formed smaller lesions on soybean and were unable to suppress host-derived reactive oxygen species (ROS). Strikingly, chemical inhibition of ROS partially restored the virulence of the *PsMTA1* mutant, indicating that m^6^A modifications are essential for neutralizing oxidative host defenses [88]. Further reinforcing the universal role of m^6^A in fungal virulence, *Cryphonectria parasitica*, the causative agent of chestnut blight, also depends on m^6^A machinery for pathogenicity. Deletion of the m^6^A methyltransferase *CpMTA1* resulted in markedly reduced disease symptoms on both chestnut stems and apples. Integrative m^6^A-seq and RNA-seq identified *CpAphA*, an acid phosphatase gene, as a direct downstream target of *CpMTA1*. Site-specific mutations at m^6^A-modified adenosines (A1306C and A1341C) within *CpAphA* significantly reduced its function and virulence, while mutations at non-methylated positions had no effect, emphasizing the functional impact of m^6^A marks [89]. Complementing the role of methylation, demethylation processes are equally vital for fungal virulence. In *C. parasitica*, deletion of the m^6^A demethylase *CpALKBH* resulted in elevated global m^6^A levels and impaired fungal growth, sporulation, and virulence. *CpALKBH* was shown to stabilize *CpZap1*, a transcription factor critical for infection, through site-specific demethylation at A1935. While overexpression of *CpZap1* in the *CpALKBH*-null context restored fungal growth and pathogenicity, loss of *CpZap1* reproduced the *CpALKBH* mutant phenotype. Virulence assays on apples and chestnut stems confirmed the functional importance of the *CpZap1* and *CpALKBH* genes. These findings establish *CpALKBH* as a critical m^6^A demethylase in *C. parasitica*, regulating virulence through m^6^A-mediated control of *CpZap1* mRNA stability [90]. Collectively, these studies reveal that m^6^A-mediated methylation and demethylation are integral components of the epitranscriptomic toolkit used by fungal and oomycete pathogens to modulate infection-related gene expression, thereby enhancing their ability to colonize and cause disease in plant hosts.

## 4. m^6^A RNA Modifications During Bacterial Infections

m^6^A is increasingly recognized as a key regulator of plant immune responses to bacterial pathogens. This dynamic and reversible epitranscriptomic mark fine-tunes mRNA metabolism, including stability, splicing, translation, and degradation, thereby shaping transcriptomic landscapes during biotic stress. Emerging studies from diverse plant systems reveal their multifaceted involvement in both basal and induced immune responses.

**Figure 3:**
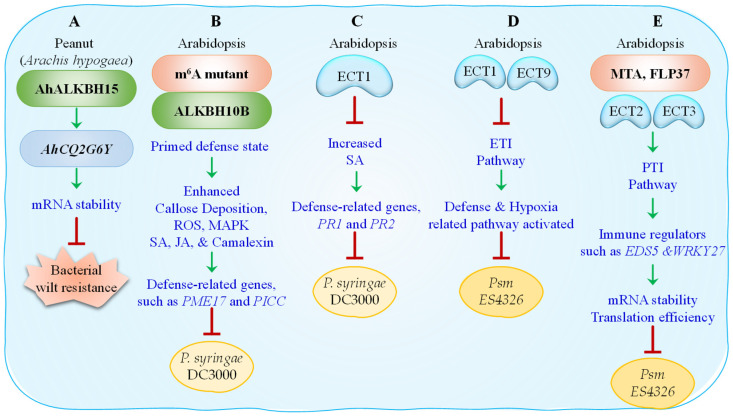
This figure shows that m^6^A RNA methylation enhances plant immunity against bacterial pathogens by regulating transcript stability, translation, and defense signaling. In peanut, AhALKBH15 stabilizes resistance genes to combat bacterial wilt. The plant m^6^A machinery components such as ALKBH10B, ECT1, ECT9, MTAs, and FLP37 boost immune-related pathways including callose deposition, ROS, MAPK, SA/JA signaling, and defense gene expression, thereby strengthening PTI and ETI pathways and restricting bacterial infections.

In peanut (*Arachis hypogaea*), resistance to bacterial wilt caused by *Ralstonia solanacearum* was linked to specific m^6^A methylation patterns (Figure 3A). Comparative analysis between resistant (H108) and susceptible (H107) lines uncovered widespread m^6^A remodeling, with a preference for methylation within 3′ untranslated regions (3′ UTRs) and enrichment of the conserved ‘URUAY’ motif. The resistant line displayed strong upregulation of genes encoding S-adenosylmethionine (SAM) synthases (*AhSAM1/2*), m^6^A writers (*AhMTA1/2/4/5*), erasers (*AhALKBH2/14/15/18*), and readers (*AhECT6–10*, *AhECT13*). Integrated m^6^A-seq and RNA-seq analyses linked these methylation changes to altered gene expression in key immune pathways. Notably, the demethylase AhALKBH15, harboring a unique coiled-helix N-terminal domain, was found to specifically demethylate the defense gene *AhCQ2G6Y*, enhancing its mRNA stability and expression. Although AhCQ2G6Y exhibited lower m^6^A levels in H108 compared to H107, its elevated expression conferred increased resistance, and its overexpression suppressed pathogen proliferation [91]. Further, a study in Arabidopsis elucidates the immunomodulatory role of m^6^A. Studies in Arabidopsis have demonstrated that plants deficient in m^6^A RNA methylation exhibit enhanced resistance to bacterial pathogens (Figure 3B). For instance, infections by *Pseudomonas syringae* pv. *tomato* DC3000 and *P. syringae* pv. *maculicola* ES4326 (Psm ES4326) were more effectively restricted in *mta* mutant plants, supporting that m^6^A plays a critical role in modulating defense responses [80]. Additional insights were gained by examining additional m^6^A-deficient lines, including *fip37-4* and *vir-2* along with *mta* mutant, as well as plants overexpressing the demethylase *ALKBH10B*, which showed enhanced resistance to *P. syringae* DC3000, together with elevated basal defense responses, including increased callose deposition, ROS production, MAPK activation, and accumulation of salicylic acid (SA), jasmonic acid (JA), and camalexin. These m^6^A-deficient plants exhibited a pre-infection enrichment of immune transcripts, indicative of a primed defense state. Further transcriptomic and m^6^A-methylome analyses revealed dynamic m^6^A redistribution, particularly within 3′ UTRs, upon pathogen challenge. Several defense-related genes, such as *PME17* (Pectin methylesterase 17) and *PICC* (PAMP-Induced Coiled Coil), showed increased expression in the absence of m^6^A, while transcripts like *CPL3*, *SCREW3*, and *VAD1* were destabilized post-elicitation, suggesting that m^6^A both represses and stabilizes immune transcripts in a context-dependent manner [80].

Further elaborating on the regulatory complexity, ECT family reader proteins fine-tune immune responses through transcript fate determination. For instance, ECT1 functions as a negative regulator of SA-mediated immunity by forming cytoplasmic condensates (Figure 3C). Upon SA treatment or infection with *P. syringae* DC3000, ECT1 undergoes liquid–liquid phase separation through its prion-like N-terminal domain, sequestering and degrading SA-induced, m^6^A-modified transcripts such as *PR1* and *PR2*. Loss of ECT1 confers SA hypersensitivity and elevated expression of SA-responsive genes, whereas its overexpression dampens immunity, confirming ECT1’s role as a negative regulator of SA-mediated defense [92]. Adding another layer of regulation, a recent study demonstrated that ECT1 and ECT9 act redundantly to suppress ETI in *Arabidopsis* (Figure 3D). While single mutants showed no significant phenotype, the *ect1*/*ect9* double mutant exhibited enhanced resistance to the avirulent strain *Psm* ES4326 (AvrRpt2), with reduced bacterial proliferation and increased cell death. Transcriptomic profiling revealed over 3800 DEGs in mock conditions and 668 DEGs during ETI, with a shared core of 389 upregulated genes enriched in defense and hypoxia-related pathways, showing their joint role in fine-tuning ETI responses [93].

A recent study has shown that m^6^A methylation is crucial not only for basal immunity but also for orchestrating pattern-triggered immunity in plants (Figure 3E). The *fip37-4* mutant and inducible MTA knockdown lines showed compromised resistance to *Hyaloperonospora arabidopsidis* and *P. syringae*, underscoring m^6^A’s central role in pathogen defense. While global m^6^A levels remained stable after peptide fragment of a bacterial protein elf18 treatment, PTI triggered transcript-specific m^6^A remodeling, particularly on immune regulators such as *EDS5* and *WRKY27*. Reader proteins ECT2/3/4 coordinated the fate of these mRNAs: ECT2 promoted translation, ECT3 stabilized transcripts, and ECT1 facilitated degradation. Mutants lacking all three readers ECT2/3/4 exhibited impaired PTI, demonstrating their cooperative roles in mRNA fate determination. Furthermore, decay of elf18-induced transcripts was delayed in *fip37-4*, and translation efficiency of ECT2-bound mRNAs was reduced in both *fip37-4* and *ect2/3/4* mutants. These results highlight m^6^A as a dual regulator, balancing rapid transcript turnover and translation efficiency to fine-tune immune responses. Polysome profiling further confirmed m^6^A’s role in enhancing the translation of immune-related genes [94].

Together, these findings from both crop and model plants establish m^6^A as a central epitranscriptomic regulator of plant immunity, orchestrating transcript-specific responses to bacterial pathogens and offering new avenues for engineering disease-resistant cultivars.

## 5. m^6^A RNA Modifications During Viral Infection

Plants resist viral infections through basal immunity, RNA silencing, R gene-mediated responses, and systemic acquired resistance. Hormonal and epigenetic regulation further fine-tune these responses, ensuring a timely and effective defense against viral pathogens [95,96,97]. m^6^A is emerging as a critical layer of epitranscriptomic regulation in virus–host interactions across diverse organisms [98]. Several transcriptome-wide studies have uncovered its role in modulating host defense gene expression and influencing viral replication. For instance, It is reported that distinct m^6^A methylation landscapes in two wheat varieties with contrasting resistance to wheat yellow mosaic virus (WYMV), identifying over 25,000 m^6^A peaks enriched at 3′ UTRs and stop codons [57]. Integration of m^6^A-seq and RNA-seq revealed that 729 genes displayed coordinated changes in methylation and expression, many involved in defense signaling, plant–pathogen interactions, protein phosphorylation, and ABA signaling. Quantitative PCR confirmed that differential m^6^A methylation in several defense-related genes, such as *TraesCS1B02G175900* (cysteine-rich receptor-like protein kinase), *TraesCS7B02G446900* (*O. sativa* GRP94 homolog), and *TraesCS7A02G267400* (PTI1-like tyrosine protein kinase 3), is likely due to aberrant expression of key m^6^A-modifying enzymes between resistant and sensitive wheat groups. These changes are probably due to altered expression of the m^6^A writer *TaFIP37-1* and eraser *TaALKBH29B*, suggesting a regulatory role in modulating m^6^A levels during viral infection [57]. Similarly, A study profiled that the m^6^A methylome in watermelon infected with cucumber green mottle mosaic virus, finding a global reduction in m^6^A levels in resistant genotypes, coinciding with demethylase gene *ClALKBH4B* upregulation. m^6^A-seq identified 422 differentially methylated genes, predominantly hypomethylated, with 59 showing coordinated changes in methylation and expression, including several associated with plant immunity [99]. In rice, It is observed that increased m^6^A levels during rice stripe virus (RSV) and rice black-streaked dwarf virus (RBSDV) infections, particularly in telomeric regions, defense-related genes, and viral RNAs, suggesting a role in host immune suppression [100]. Genome-wide m^6^A profiling in rice infected with RBSDV and RSV identified over 20,000 unique m^6^A peaks per treatment, including virus-specific and shared sites. Notably, m^6^A was predominantly enriched in lowly expressed genes and those regulating key components of RNA silencing and hormone signaling pathways (JA, SA, ABA, auxin, CTK, ET, and BR), indicating its involvement in broader stress responses. KEGG analysis revealed enrichment in defense and metabolic pathways. Antiviral genes such as *OsAGO18* and *OsSLRL1* exhibited expression changes correlated with m^6^A enrichment. qRT-PCR further confirmed RSV-induced upregulation of *OsMAT3/4* and consistent downregulation of *OsALKBH10* by both viruses [100]. Another recent study demonstrates that Prunus necrotic ringspot virus (PNRSV) reshapes the m^6^A RNA modification landscape in *Cucumis sativus*, affecting immunity and metabolism. Transcriptome and epitranscriptome analyses showed upregulation of m^6^A writers (*CuMTA*, *CuMTB*, *CuHAKAI*) and methyl donors (*CuSAM2a*, *CuSAM4*), along with downregulation of the eraser *CuALKBH10B* and readers (*CuECT2*, *CuECT4a*, *CuECT4b*), indicating disrupted m^6^A homeostasis. KEGG enrichment of differentially methylated genes highlighted pathways in glyoxylate and dicarboxylate metabolism, motor function, and cyanoamino acid metabolism. Notably, the defense gene *CuPAL* was hypermethylated and transcriptionally induced during infection, and its silencing increased viral load [101]. Collectively, these studies show that plants modulate m^6^A methylation in response to viral infection, regulating immune and stress-related genes. Transcriptome-wide profiling highlights m^6^A’s role in antiviral defense and its potential as a target for enhancing disease resistance.

The interplay between m^6^A methyltransferases and demethylases significantly influences viral RNA stability and accumulation. A study identified *Triticum aestivum* m^6^A methyltransferase B (TaMTB) as a positive regulator of WYMV infection in wheat. TaMTB binds to the WYMV NIb protein, promoting m^6^A deposition and stabilizing viral RNA1, thereby enhancing infection. Moreover, a natural allele, TaMTB-SNP176C, was found to increase susceptibility across 243 wheat cultivars, underlining its agronomic relevance [102]. In contrast, *Tobacco Mosaic Virus* (TMV) infection was associated in *Nicotiana tabacum* with significant reductions in global m^6^A levels. Using UHPLC-HR-MS/MS, It is identified that upregulation of a putative ALKBH5-like demethylase (XM_009801708) and downregulation of methyltransferase genes. This suggests that TMV manipulates host m^6^A machinery by promoting demethylase expression, potentially disrupting normal RNA regulation [103]. Further, He et al. (2023) showed that *Pepino Mosaic Virus* (PepMV) evades m^6^A-mediated defense by promoting autophagic degradation of the m^6^A writer SlHAKAI in tomato [104]. The viral RNA-dependent RNA polymerase interacts with both SlHAKAI and the autophagy protein SlBeclin1, triggering SlHAKAI degradation. Overexpression of *SlHAKAI* reduces PepMV RNA and protein levels via m^6^A modification, while autophagy inhibition or Beclin1 silencing blocks this degradation. These findings reveal that PepMV hijacks the autophagy pathway to suppress m^6^A-dependent antiviral immunity [104]. Similarly, a recent study investigated the role of methyltransferase-like (METTL) proteins in *Nicotiana benthamiana* during infection with *plum pox virus* (PPV). Transcriptomic analysis revealed significant downregulation of *METTL* genes, and subsequent cloning of *NbMETTL1* and *NbMETTL2* confirmed their homology to human *METTL16* and Arabidopsis *FIONA1*, with conserved SAM-binding domains. Overexpression of these *METTLs* significantly reduced PPV accumulation, suggesting their role in epitranscriptomic antiviral defense [105]. Likewise, overexpression of *Phaseolus vulgaris PvMTA* inhibits bean common mosaic virus infection via m^6^A-mediated mechanisms, whereas silencing *PvMTA* enhances susceptibility [106]. These contrasting strategies highlight how viruses either exploit or suppress m^6^A writers to manipulate host cellular machinery, illustrating the versatile and reversible nature of m^6^A as an epigenetic mechanism in plant–virus interactions.

A growing body of evidence implicates the AlkB family of m^6^A demethylases as pivotal modulators of plant antiviral responses, with some members acting as proviral factors. In *Arabidopsis*, m^6^A demethylase AtALKBH9B was identified to promote *alfalfa mosaic virus* (AMV) infection by removing methyl groups from viral RNAs, thereby facilitating replication and movement. While AtALKBH9B colocalizes with siRNA bodies and P bodies, its demethylation activity affects AMV but not *Cucumber mosaic virus* (CMV), likely due to interactions with viral coat proteins [107]. Further studies confirmed that only ALKBH9B, and not its homologs ALKBH9A or 9C, is essential for AMV replication and systemic spread, particularly during early infection stages and blocks viral phloem loading [108]. Interestingly, YTH-domain m^6^A reader proteins (ECT2, ECT3, ECT5) counteracted this proviral effect; disruption of these readers restored AMV infectivity in *alkbh9b* mutants, revealing an antiviral role and the functional balance between m^6^A writing, erasing, and reading in plant–virus interactions [109]. Expanding on this, a recent study identified AlkB domains in the *Endive necrotic mosaic virus* P1 protein and detected similar motifs in a *Potyvirus*. Interestingly, infection by AlkB-lacking *Potyviruses* (e.g., *plum pox virus* (PPV) and *potato virus Y* (PVY)) led to reduced m^6^A levels in *Nicotiana benthamiana*, with m^6^A enrichment observed in host 3′ UTRs and viral genomes. Silencing *N. benthamiana AlkB* homologs significantly reduced viral (PPV and PVY) accumulation, suggesting that both host and viral AlkB domains contribute to *Potyvirus* infection and evolution [110]. Further, A recent study in *Brassica juncea* revealed that the m^6^A demethylase BjALKBH9B interacts with both *Turnip mosaic virus* (TuMV) and the eukaryotic translation initiation factor eIF2Bβ. Eukaryotic initiation factors (eIFs) are known targets for engineering plant RNA virus resistance. Genome editing of *eIF2Bβ* enhanced TuMV resistance in both *B. juncea* and *B. napus*. Furthermore, BjALKBH9B was shown to interact with BjeIF2Bβ and TuMV, modifying the m^6^A status of TuMV coat protein RNA to regulate infection. These findings not only identify eIF2Bβ as a promising target for virus-resistant breeding but also reveal a non-canonical mechanism of translational control in plant–virus interactions [111]. These studies reveal that m^6^A demethylases regulate both host and viral RNAs and are exploited by viruses to enhance infection, making them key targets for developing virus-resistant crops.

In addition to writers and erasers, m^6^A readers also play crucial roles in shaping plant antiviral defense. it has been demonstrated that m^6^A readers such as ECT2, ECT3, and ECT5 act downstream of the demethylase ALKBH9B to restrict AMV infectivity in Arabidopsis. These YTH domain proteins recognize methylated viral RNAs and mediate downstream regulatory effects that limit viral propagation [109]. Complementing these findings, A study investigated m^6^A dynamics in *N. benthamiana* and tomato during PepMV infection and found that overexpression of m^6^A writers (*MTA*, *HAKAI*) suppressed viral accumulation, while loss of function enhanced infection. Notably, YTH domain-containing proteins (NbECT2A/2B/2C) functioned as m^6^A readers and, together with nonsense-mediated decay (NMD) components NbUPF3 and NbSMG7, promoted viral RNA degradation [112]. A recent study further supports this, showing that PNRSV infection in *Cucumis sativus* significantly reprograms the host m^6^A methylation landscape. Silencing of m^6^A readers *CuECT2* and *CuECT4a/4b*, leading to elevated PNRSV RNA and coat protein levels. Similarly, knockdown of key components of the NMD pathway, *CuUPF3* and *CuSMG7*, further increased viral accumulation [101]. These findings provide new insights into a functional cooperation between m^6^A reader proteins that cooperate with RNA decay pathways to suppress viral infection, revealing a novel layer of RNA-based plant immunity.

The role of m^6^A methylation extends beyond the plant host and includes the insect vectors responsible for virus transmission.) It is investigated that the m^6^A methylation landscape in the midgut of the small brown planthopper (*Laodelphax striatellus*) following infection with Rice black-streaked dwarf virus (RBSDV). The study found that virus infection led to a significant reduction in m^6^A levels in the insect midgut. Silencing the m^6^A methyltransferase genes *LsMETTL3* and *LsMETTL14* resulted in increased viral accumulation, indicating that m^6^A methylation serves as an antiviral mechanism in insect vectors as well [113]. These findings suggest that m^6^A-mediated antiviral defense is conserved across kingdoms and may play a universal role in host–pathogen interactions.

**Figure 4:**
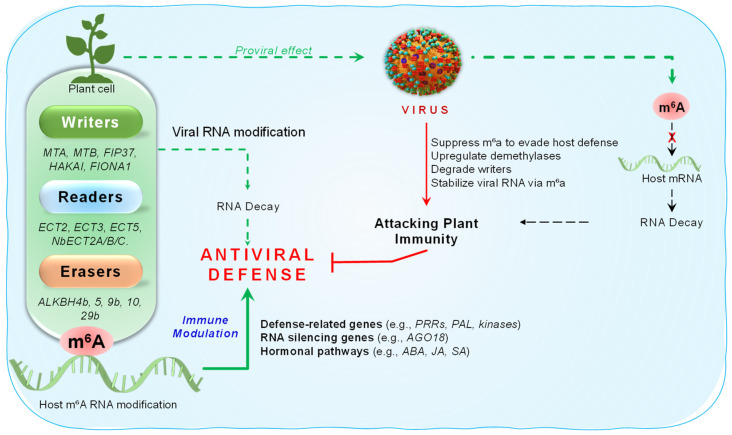
The illustration shows how m^6^A alterations act as a double-edged sword across several levels, protecting against and being exploited by viruses.

The interplay between m^6^A RNA modifications and host–virus interactions has emerged as a critical aspect of plant antiviral defense, with both host enzymes and viral RNA structures contributing to infection outcomes. In Arabidopsis, the m^6^A demethylase AtALKBH9B interacts with the coat protein (CP) of AMV, thereby modulating viral infection. Functional dissection of AtALKBH9B revealed that amino acid residues 427–467 are essential for AMV RNA binding, while residues 387–427 mediate direct interaction with the CP. Additionally, the protein contains intrinsically disordered regions (IDRs), including a C-terminal RGxxxRGG RNA-binding motif, which are critical for its function. Deletion of the N-terminal 20 or C-terminal 40 residues impaired its localization to siRNA bodies. These findings identify crucial regions of AtALKBH9B required for RNA binding, viral interaction, and subcellular localization during AMV infection [114]. Complementing these findings, recent research has shown that not only host proteins but also the structural context of viral RNA itself significantly influences m^6^A methylation and viral replication. A comprehensive analysis of the 3′ UTR of AMV RNA3 identified two conserved hairpin structures (hpB and hpE) crucial for viral RNA accumulation. These elements were found to facilitate the accumulation of plus-strand RNA during the early stages of AMV infection, acting as cis-regulatory scaffolds that guide m^6^A deposition to functionally important sites. This structural modulation of epitranscriptomic marks underscores the sophisticated level at which viruses exploit RNA architecture to fine-tune replication [115]. Together, these studies reveal a finely balanced relationship between host m^6^A regulatory proteins and viral RNA structures, emphasizing how both molecular players co-evolve to shape infection dynamics and providing novel targets for engineering virus resistance in plants (Figure 4).

## 6. m^6^A RNA Modifications During Insect and Nematode Infestations

Plant resistance to insect and nematode infestations involves complex defense mechanisms, including the activation of resistance genes, hormone signaling pathways like jasmonic acid, salicylic acid, and ethylene, and transcriptional reprogramming [116,117,118,119,120]. These responses regulate defense-related proteins, secondary metabolites, and cell wall modifications to restrict pest invasion and minimize damage [121,122]. m^6^A RNA methylation, a dynamic and reversible epitranscriptomic modification, is emerging as a key regulator of plant responses to biotic stress, including insect attacks, although its role in plant–insect interactions remain incompletely understood. Recent studies across multiple plant systems have begun to elucidate its functional significance in insect resistance. In rice, *Nilaparvata lugens* (brown planthopper) infestation was shown to enhance host resistance while concurrently reducing global m^6^A methylation levels. Interestingly, defense-related pathways such as those mediated by jasmonic acid and salicylic acid exhibited increased m^6^A modification, whereas growth-associated pathways, including auxin and gibberellin biosynthesis, showed decreased methylation. This shift reflects a strategic reallocation of resources from growth to defense, with a positive correlation observed between m^6^A levels and gene expression in defense pathways [123]. In a complementary study focusing on rice resistance to the striped stem borer (RSB), larvae exhibited more robust growth on indica varieties (MH63, IR64, Nanjing 11) than on japonica types (Nipponbare, Zhonghua 11, Xiushui 11). Using nanopore direct RNA sequencing, researchers found that RSB infestation led to an overall reduction in m^6^A methylation across the genome but retained enrichment in actively transcribed genes. In the resistant japonica cultivar Nipponbare, enhanced m^6^A methylation and expression were observed in key defense pathways, including mitogen-activated protein kinase cascades, jasmonate biosynthesis, and terpenoid metabolism. Notably, trypsin protease inhibitor genes involved in jasmonate-mediated defense exhibited strong m^6^A enrichment, suggesting a role for m^6^A in fortifying insect resistance [124]. Extending these insights to woody species, a recent investigation into *Pinus massoniana* uncovered 22 m^6^A regulatory genes, comprising 7 writers, 7 erasers, and 8 readers. Expression analysis in clones with contrasting resistance to *Monochamus alternatus* revealed differential regulation of 14 m^6^A factors, with *PmALKBH3*, *PmYTHDF5*, and *PmHAKAI1* downregulated in susceptible clones, while *PmMTA*, *PmMTB*, *PmHAKAI2*, and *PmYTHDF1–3* was notably upregulated in resistant ones. These findings suggest a context-specific role of m^6^A methylation in orchestrating plant immunity against insect herbivores across diverse plant taxa and point toward molecular targets for developing insect-resistant crop and forest species [125]. Collectively, these findings underscore the crucial and context-specific role of m^6^A in plant defense against insect herbivores and provide promising epitranscriptomic targets for breeding pest-resistant crop and forest species.

Emerging evidence highlights the critical role of m^6^A RNA methylation in regulating plant defense responses against nematodes. This dynamic epitranscriptomic modification influences gene expression, metabolite biosynthesis, and immune signaling during nematode infection, as demonstrated in soybean. A comprehensive analysis of *Meloidogyne incognita*-infected soybean roots revealed widespread m^6^A hypermethylation, particularly at 3′ UTRs. The study identified 2069 altered m^6^A sites, 594 differentially expressed genes (DEGs), and 103 metabolite changes. Flavonoid biosynthesis appeared to be epitranscriptomically regulated, with significant accumulation of coumestrol and psoralidin in infected roots, likely driven by m^6^A-modified MYB transcription factor. Additionally, two ROS-related genes, *BBE-like 28* and *POD47*, were hypomethylated and transcriptionally upregulated, indicating a possible role in early ROS-mediated basal defense near nematode feeding sites. With the exception of *ERF60*, all examined transcription factors, including *WRKY70*, *HSF A7a*, *MYB114*, *MYB124*, and *ZFP*, showed an inverse relationship between m^6^A methylation and expression, as validated by m^6^A-IP-qPCR and qRT-PCR. Other differentially expressed defense-related proteins included lectin and leucine-rich repeat (LRR) receptor kinases, cytochrome P450s, and components of the ubiquitin–proteasome system. Collectively, these findings suggest that m^6^A methylation orchestrates a multifaceted defense strategy against *M. incognita* by modulating transcript stability, secondary metabolism, and immune signaling [126]. Another related study investigated m^6^A methylation in soybean resistance to another economically important nematode, *Heterodera glycines*. By comparing resistant (11452) and susceptible (DS1) breeding lines, researchers uncovered striking differences in m^6^A methylation and gene expression at three days post-inoculation, which were critical for early nematode infestation. In the resistant genotype, defense-related genes such as resistance genes, receptor-like kinases, and transcription factors were upregulated alongside reduced m^6^A methylation, suggesting a derepression mechanism in which hypomethylation facilitates immune activation. Conversely, the susceptible genotype exhibited elevated expression of susceptibility-associated genes, such as *MLO*-like proteins and other negative regulators of defense, which were also hypomethylated. These contrasting patterns indicate the dual role of m^6^A as both a positive and negative regulator of plant immunity, depending on the function of the target gene. Further analysis revealed that m^6^A acts in coordination with alternative splicing, transcriptional control, and small RNA pathways, forming an integrated defense network. This study highlights m^6^A’s role in early immune signaling pathways during pathogen response [127]. These insights not only advance our understanding of epitranscriptomic regulation in plant nematode interactions but also identify promising targets for developing soybean cultivars with enhanced resistance to nematode infestations.

A summary of all genes involved in plant defense against fungi, bacteria, viruses, insects, and nematodes regulated by m^6^A-mediated plant immunity has been provided in Table 1.

## 7. Limitations and Future Perspectives

Despite substantial progress in decoding the role of m^6^A modifications in plant–pathogen interactions, several critical gaps remain. Much of the current understanding stems from studies conducted on a limited number of model species, namely, Arabidopsis, soybean, and rice, leaving a significant knowledge gap regarding m^6^A function in other economically important crops. Moreover, the molecular determinants that govern the site-specific deposition and recognition of m^6^A under biotic stress are still largely undefined, limiting our understanding of how this modification is dynamically regulated in different biological contexts. In addition to these taxonomic and mechanistic gaps, many investigations primarily rely on high-throughput correlative data derived from m^6^A-seq and RNA-seq analyses. While these approaches are valuable for identifying potential targets and patterns, they often lack direct molecular or biochemical validation, making it difficult to definitively link observed methylation changes to functional outcomes. This discrepancy emphasizes the necessity of more functional research to elucidate the specific functions of m^6^A in plant immunity. Moving forward, addressing these limitations will require an integrated approach. Future research should investigate the interplay between m^6^A and other layers of gene regulation, including alternative splicing, small RNAs, Transcription factors, RNA polymerase, and chromatin modifications, to understand how these networks converge during stress responses. Moreover, advanced genome editing tools such as CRISPR/Cas systems present powerful opportunities to manipulate specific components of the m^6^A machinery with high precision. Under pathogen challenge, these techniques may be used to analyze the phenotypic effects of gene-specific functions. Equally important is the translation of laboratory findings to field conditions. By combining epitranscriptomic knowledge with plant breeding and synthetic biology, it may become possible to engineer crops with enhanced, durable resistance to pathogens. Such advances will be vital for developing sustainable and resilient agricultural systems in the face of mounting biotic stresses.

## 8. Conclusions

The rapidly advancing field of plant epitranscriptomics, with m^6^A mRNA modification at its core, is fundamentally reshaping our understanding of RNA-based regulatory mechanisms in plant defense. As a dynamic and reversible RNA modification, m^6^A acts as a versatile regulator of mRNA metabolism, modulating transcript stability, splicing, translation, and degradation in a highly context-dependent manner. Importantly, both plant hosts and their invading pathogens have evolved strategies to reprogram m^6^A methylation landscapes, thereby influencing the outcome of infection and defense. The conserved nature of m^6^A across species, coupled with its regulatory plasticity, highlights its potential as a strategic target for enhancing crop resilience. With mounting evidence linking m^6^A to the fine-tuning of immune responses, it is increasingly viewed as a promising molecular lever for engineering disease-resistant varieties. Moving forward, unravelling the mechanistic details and functional consequences of m^6^A modifications, particularly in agronomically important crops, will be critical. Ultimately, integrating m6A-focused insights with cutting-edge genomic, transcriptomic, and gene editing tools will pave the way for innovative and sustainable strategies in crop protection. As global agriculture faces intensifying biotic challenges, harnessing epitranscriptomic regulation through m^6^A offers a forward-looking solution to safeguard productivity and food security.

## Figures and Tables

**Table 1:** Summary of Genes Involved in m^6^A-Mediated Plant Immunity during different biotic stresses.

Gene #	Organism	Type/Role	Function in Immunity/Virulence	Pathogen Context
FUNGUS INFECTION				
*MTA*	*Arabidopsis*	Writer	*mta* mutants show stronger resistance; m^6^A normally reduces defense signaling	*Botrytis cinerea*
*HAKAI1*	*Arabidopsis*	Writer	*hakai-1* mutants exhibit constitutive basal defense and enhanced resistance	*Hyaloperonospora arabidopsidis*
*MdVIR1*, *MdVIR2*	*Malus domestica* (apple)	Writers	Enhance defense	*Alternaria alternata*
*MdWRKY79*	*M. domestica*	Transcription factor (TF)	Stabilized by m^6^A, promoting immune response	*A. alternata*
*MdNLR16*	*M. domestica*	NLR resistance	Stabilized by m^6^A, enhancing immunity	*A. alternata*
*TaALKBH11B*	*Triticum aestivum* (wheat)	Eraser	Upregulated during early infection	*Puccinia striiformis* f. sp. tritici (Pst)
*TaALKBH4B*	*T. aestivum*	Eraser	Peaks during colonization	*Pst*
*TaECT25*, *TaECT31*, *TaECT21*	*T. aestivum*	Readers	Downregulated at early infection	*Pst*
*TaVIR-D*, *TaVIR-A*, *TaHAKAI1-A*	*T. aestivum*	Writers	Reduced activity during infection	*Pst*
*TaFIP37-2D*	*T. aestivum*	Writer	Continuously induced; central in defense regulation	*Pst*
*MTA*, *ALKBH5B*, *ECT9*	*Pyrus communis* (pear)	Writer, Eraser, Reader	Core network regulating PTI and SAR immune genes	*Erwinia amylovora*
*WRKYs*, *Ser/Thr kinases*	*P. communis*	Defense genes	m^6^A-modified, positively correlated with higher expression	*E. amylovora*
*MhYTP2*	*Malus hupehensis*	Reader (ECT2 homolog)	Enhances resistance to *P. leucotricha*;	*Podosphaera leucotricha*, *Colletotrichum fructicola*
*MdMTA*, *MdMTB*, *MdFIP37*	*M. domestica*	Writers	Downregulated by MhYTP2 overexpression	*P. leucotricha*
*MdALKBH6*	*M. domestica*	Eraser	Downregulated by MhYTP2 overexpression	*P. leucotricha*
*MdMLO19*, *MdMLO19-X1*	*M. domestica*	Susceptibility genes	Downregulated via m^6^A-mediated degradation	*P. leucotricha*
*MdGDH1L*	*M. domestica*	Antioxidant enzyme	m^6^A enhances translation → higher antioxidant activity	*P. leucotricha*
*MdRGA2L*	*M. domestica*	NBS-LRR resistance	Promotes SA biosynthesis via ICS1; suppressed by MhYTP2	*C. fructicola*
BACTERIAL INFECTION				
*AhSAM1/2*	*Arachis hypogaea*	SAM synthases	Provide methyl donor for m^6^A; upregulated in resistant line H108	*Ralstonia solanacearum* (bacterial wilt)
*AhMTA1/2/4/5*	*A. hypogaea*	Writers	Core m^6^A methyltransferases; upregulated in resistant line	*R. solanacearum*
*AhALKBH2/14/15/18*	*A. hypogaea*	Erasers	Dynamic regulation of defense transcripts	*R. solanacearum*
*AhALKBH15*	*A. hypogaea*	Eraser	demethylates defense gene AhCQ2G6Y, enhancing stability and resistance	*R. solanacearum*
*AhECT6–10*, *AhECT13*	*A. hypogaea*	Readers	Bind m^6^A-modified RNAs; regulate immune transcript fate	*R. solanacearum*
*AhCQ2G6Y*	*A. hypogaea*	Defense gene	Stabilized by AhALKBH15 demethylation; elevated expression suppresses pathogen growth	*R. solanacearum*
*MTA*	Arabidopsis	Writer	*mta* mutants show enhanced resistance;	*Pseudomonas syringae pv. tomato DC3000*, *Psm ES4326*
*FIP37-4*	Arabidopsis	Writer subunit	Mutants show enhanced resistance; impaired PTI; delayed transcript decay	*P. syringae* DC3000, elf18 treatment
*VIR-2*	Arabidopsis	Writer subunit	Enhances resistance	*P. syringae*
*ALKBH10B*	Arabidopsis	Eraser	Overexpression enhances resistance; increases basal defenses (ROS, callose, SA, JA, camalexin)	*P. syringae* DC3000
*PME17*, *PICC*	Arabidopsis	Defense genes	regulatory protein PICC role in callose deposition; Show higher expression in absence of m^6^A,	*P. syringae*
*CPL3*, *SCREW3*, *VAD1*	Arabidopsis	Defense-related genes	Destabilized post-elicitation in m^6^A-deficient plants	*P. syringae*
*ECT1*	Arabidopsis	Reader	Negative regulator of SA-mediated defense; forms condensates to degrade SA-induced PR1/PR2 transcripts	*P. syringae* DC3000
*PR1*, *PR2*	Arabidopsis	SA-responsive defense genes	m^6^A-modified; degraded by ECT1 to suppress overactive immunity	*P. syringae* DC3000
*ECT9*	Arabidopsis	Reader	Redundant with ECT1; suppresses ETI	*Psm* ES4326 (AvrRpt2)
*ECT1/9*	Arabidopsis	Readers	Double mutant shows enhanced ETI, reduced pathogen growth, increased cell death	*Psm* ES4326 (AvrRpt2)
*EDS5*	Arabidopsis	SA pathway regulator	Shows PTI-associated transcript-specific m^6^A remodeling	elf18 treatment
*WRKY27*	Arabidopsis	TF (immune regulator)	Target of m^6^A remodeling during PTI	elf18 treatment
*ECT2*	Arabidopsis	Reader	Promotes translation of immune transcripts (e.g., WRKY27, EDS5)	elf18 treatment, *P. syringae*
*ECT3*	Arabidopsis	Reader	Stabilizes immune transcripts	elf18 treatment
*ECT4*	Arabidopsis	Reader	Works with ECT2/3 in cooperative PTI regulation	elf18 treatment
VIRAL INFECTION				
*TraesCS1B02G175900*	*T. aestivum*	Defense kinase	Differentially methylated; cysteine-rich receptor-like protein kinase in defense signaling	WYMV
*TraesCS7B02G446900*	*T. aestivum*	Defense chaperone	GRP94 homolog; methylation linked to defense regulation	WYMV
*TraesCS7A02G267400*	*T. aestivum*	Defense kinase	PTI1-like kinase; participates in PTI signaling, m^6^A-modulated	WYMV
*TaFIP37-1*	*T. aestivum*	Writer	Regulates methylation of defense genes during infection	WYMV
*TaALKBH29B*	*T. aestivum*	Eraser	Modulates methylation of immune genes	WYMV
*ClALKBH4B*	Watermelon	Eraser	Upregulated in resistant genotype; linked to reduced m^6^A, enhancing immunity	CGMMV
*OsAGO18*	Rice (*Oryza sativa*)	Antiviral gene	Expression correlates with m^6^A enrichment; regulates antiviral RNA silencing	RSV, RBSDV
*OsSLRL1*	*O. sativa*	Defense regulator	Expression linked to m^6^A changes; modulates immunity	RSV, RBSDV
*OsMAT3/4*	*O. sativa*	Writer	Upregulated during infection; enhances m^6^A deposition	RSV
*OsALKBH10*	*O. sativa*	Eraser	Downregulated during infection; linked to viral exploitation of host m^6^A	RSV, RBSDV
*CuMTA*, *CuMTB*, *CuHAKAI*	Cucumber	Writers	Upregulated; alter global m^6^A during infection	PNRSV
*CuSAM2a*, *CuSAM4*	Cucumber	SAM synthases	Provide methyl donors; upregulated in infection	PNRSV
*CuALKBH10B*	Cucumber	Eraser	Downregulated; loss of demethylation contributes to infection	PNRSV
*CuECT2*, *CuECT4a/4b*	Cucumber	Readers	Silencing increases viral RNA accumulation	PNRSV
*CuPAL*	Cucumber	Defense gene	Hyper-methylated and transcriptionally induced; silencing increases susceptibility	PNRSV
*TaMTB*	Wheat	Writer	Promotes infection by stabilizing WYMV RNA1; natural SNP variant increases susceptibility	WYMV
*NbMETTL1*, *NbMETTL2*	*N. benthamiana*	Writers (METTL-like)	Overexpression reduces PPV accumulation (antiviral)	PPV
*PvMTA*	Common bean	Writer	Overexpression inhibits BCMV; silencing enhances susceptibility	BCMV
*AtALKBH9B*	Arabidopsis	Eraser	Promotes AMV infection by demethylating viral RNA; interacts with viral coat protein	AMV
*ECT2*, *ECT3*, *ECT5*	Arabidopsis	Readers (YTH-domain)	Restrict AMV infection by recognizing methylated viral RNAs	AMV
*SlHAKAI*	Tomato	Writer	Targeted for autophagic degradation by PepMV to suppress host m^6^A immunity	PepMV
*NbECT2A/2B/2C*	*N. benthamiana*	Readers	Promote viral RNA degradation with NMD components	PepMV
*NbUPF3*, *NbSMG7*	*N. benthamiana*	NMD pathway genes	Cooperate with m^6^A readers to degrade viral RNAs	PepMV
*BjALKBH9B*	*Brassica juncea*	Eraser	Interacts with TuMV and eIF2Bβ; regulates viral RNA methylation	TuMV
*BjeIF2Bβ*	*B. juncea*	Translation factor	Genome editing enhances resistance; targeted by BjALKBH9B–TuMV complex	TuMV
*LsMETTL3*, *LsMETTL14*	Planthopper vector	Writers	Silencing increases RBSDV accumulation; antiviral role in insect vector	RBSDV
INSECT AND NEMATODES INFESTATION				
Trypsin protease inhibitor genes	*O. sativa*	Defense protein; JA-mediated	Strong m^6^A enrichment and upregulation; inhibits insect digestive enzymes, enhancing JA-mediated insect resistance	Striped stem borer (RSB)
MAPK cascade genes	*O. sativa*	Signal transduction	Enriched in resistant cultivar; m^6^A promotes activation of MAPK signaling, reinforcing immune responses	Striped stem borer (RSB)
Jasmonate biosynthesis genes	*O. sativa*	Hormone signaling	m^6^A enrichment enhances expression, boosting JA-mediated defense	Striped stem borer (RSB)
Terpenoid metabolism genes	*O. sativa*	Secondary metabolism	Enriched in resistant cultivar; supports defense via secondary metabolite accumulation	Striped stem borer (RSB)
*PmALKBH3*, *PmYTHDF5*, *PmHAKAI1*	*Pinus massoniana*	Eraser, reader, writer (susceptible clones downregulated)	Downregulation associated with susceptibility to insect infestation	*Monochamus alternatus*
*PmMTA*, *PmMTB*, *PmHAKAI2*, *PmYTHDF1–3*	*P. massoniana*	Writers/Readers (resistant clones upregulated)	Upregulation linked to enhanced resistance; context-specific m^6^A regulatory roles	*M. alternatus*
MYB TF (regulating coumestrol, psoralidin)	Soybean (*Glycine max*)	TF; metabolite regulation	m^6^A modification drives flavonoid accumulation, strengthening defense	*M. alternatus*
*BBE-like 28*, *POD47*	*G. max*	ROS-related enzymes	Hypomethylated and upregulated; enhance ROS production for basal defense near nematode feeding sites	*Meloidogyne incognita*
*WRKY70*, *HSF A7a*, *MYB114*, *MYB124*, *ZFP*, *ERF60*	*G. max*	TFs	Mostly inverse relation between m^6^A and expression; regulate immune responses	*M. incognita*
Lectin & LRR receptor kinases	*G. max*	Receptor proteins	Differentially expressed; involved in immune perception and signaling	*M. incognita*
Cytochrome P450s	*G. max*	Secondary metabolism enzymes	Modulated by m^6^A; support production of defense metabolites	*M. incognita*
Ubiquitin–proteasome system components	*G. max*	Protein degradation machinery	Differential regulation under infection; involved in immune regulation	*M. incognita*
Resistance genes, receptor-like kinases, TFs	*G. max*	Defense regulators (upregulated in resistant genotype)	Reduced m^6^A methylation enables derepression and immune activation	*Heterodera glycines*
MLO-like proteins, negative regulators	*G. max*	Susceptibility genes (upregulated in susceptible genotype)	Hypomethylated, allowing expression of susceptibility pathways	*H. glycines*

# Refer reference of each gene function in the text.

## Data Availability

Not applicable.
